# Intersection Between Large Granular Lymphocyte Leukemia and Rheumatoid Arthritis

**DOI:** 10.3389/fonc.2022.869205

**Published:** 2022-05-13

**Authors:** Katharine B. Moosic, Kusuma Ananth, Felipe Andrade, David J. Feith, Erika Darrah, Thomas P. Loughran

**Affiliations:** ^1^University of Virginia Cancer Center, University of Virginia School of Medicine, Charlottesville, VA, United States; ^2^Department of Medicine, Division of Hematology/Oncology, University of Virginia School of Medicine, Charlottesville, VA, United States; ^3^Department of Pathology, University of Virginia School of Medicine, Charlottesville, VA, United States; ^4^Department of Medicine, Division of Rheumatology, The Johns Hopkins University School of Medicine, Baltimore MD, United States

**Keywords:** rheumatoid arthritis, cytotoxic T lymphocyte (CTL), citrullination, neutropenia, STAT3 (signal transducer and activator of transcription 3), Felty syndrome

## Abstract

Large granular lymphocyte (LGL) leukemia, a rare hematologic malignancy, has long been associated with rheumatoid arthritis (RA), and the diseases share numerous common features. This review aims to outline the parallels and comparisons between the diseases as well as discuss the potential mechanisms for the relationship between LGL leukemia and RA. RA alone and in conjunction with LGL leukemia exhibits cytotoxic T-cell (CTL) expansions, HLA-DR4 enrichment, RA-associated autoantibodies, female bias, and unknown antigen specificity of associated T-cell expansions. Three possible mechanistic links between the pathogenesis of LGL leukemia and RA have been proposed, including LGL leukemia a) as a result of longstanding RA, b) as a consequence of RA treatment, or c) as a driver of RA. Several lines of evidence point towards LGL as a driver of RA. CTL involvement in RA pathogenesis is evidenced by citrullination and granzyme B cleavage that modifies the repertoire of self-protein antigens in target cells, particularly neutrophils, killed by the CTLs. Further investigations of the relationship between LGL leukemia and RA are warranted to better understand causal pathways and target antigens in order to improve the mechanistic understanding and to devise targeted therapeutic approaches for both disorders.

## LGL Leukemia Clinical Presentation and Epidemiology

Large granular lymphocyte (LGL) leukemia, is a rare hematologic malignancy accounting for 2-5% of lymphoproliferative disorders in North America and Europe (1). Recent population-based studies place the incidence of LGL leukemia between 0.2-0.72 per million people (2, 3). There are three major subtypes of disease that exhibit T-cell or natural killer (NK) cell phenotypic markers; 85% of cases are categorized as T-LGL, 10-15% as a chronic lymphoproliferative disorder of natural killer cells (CLPD-NK), and rare cases are described as aggressive NK cell leukemia (4). The median age of diagnosis is roughly 65 years (2–4).

Approximately 45-60% of patients with LGL leukemia require treatment upon presentation, with neutropenia and anemia as the main indications for treatment. Single agent immunosuppressive agents that are utilized include methotrexate, cyclophosphamide, and cyclosporine (1, 3). A “watch-and-wait” approach is appropriate in many indolent LGL leukemia patients. Unfortunately, most patients will eventually require treatment, and despite initial response, many will relapse or need life-long therapy, thus highlighting a need for continued research and new therapeutics. Reports vary in terms of survival with one of the largest population-based studies suggesting a median 9-year overall survival (3) and others indicating that overall survival is similar to control populations (2, 5). In patients requiring treatment, survival differed between symptom type, with those affected by anemia showing a median overall survival of 5.75 years and those with neutropenia exhibiting a median overall survival not yet reached 13 years after initiation of the study (6). Together, these reports demonstrate the heterogeneity of the patient population and the relatively indolent nature of the disease.

**Figure 1:**
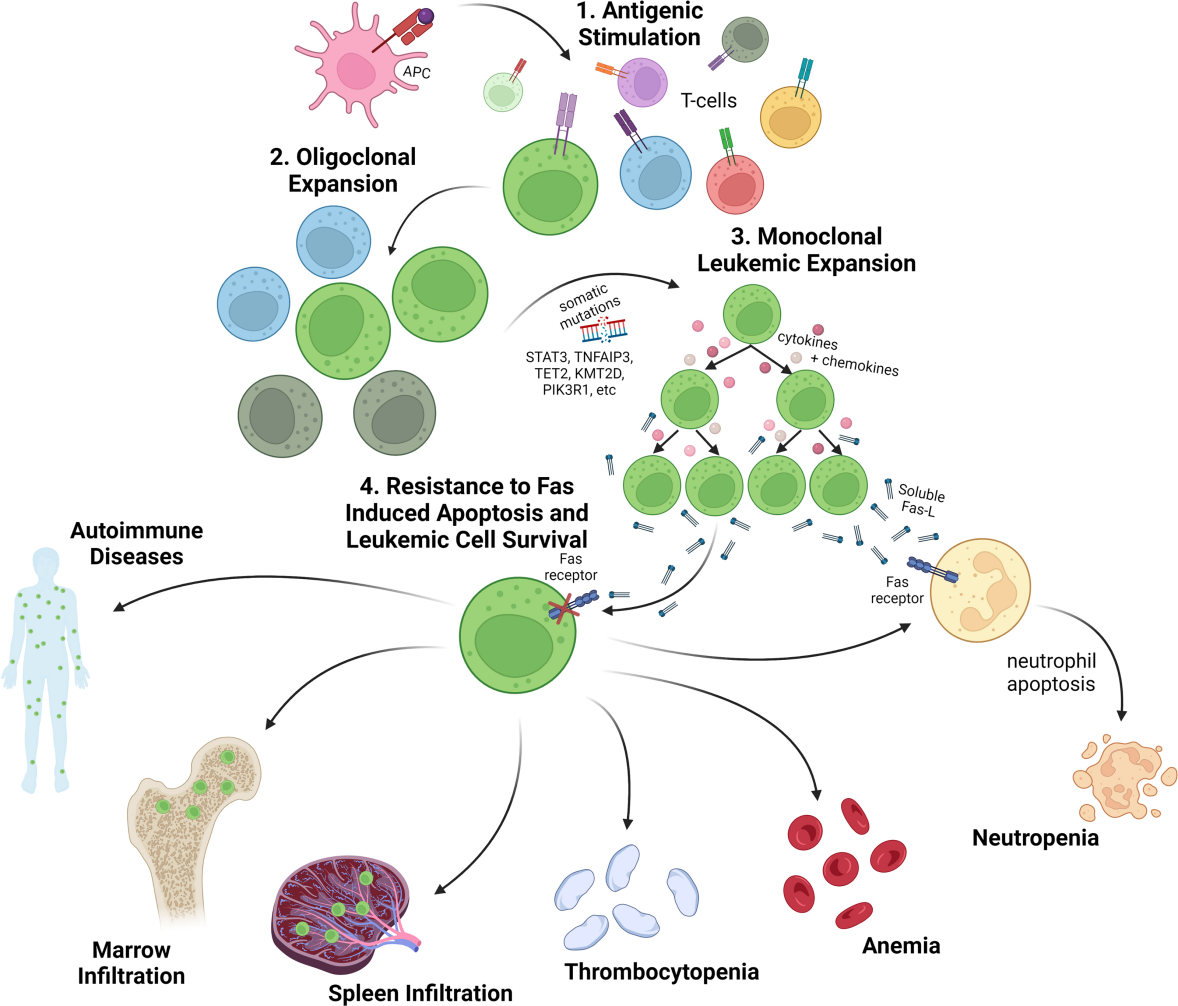
Overview of LGL leukemia pathogenesis and clinical presentation. 1. T-cell LGL leukemia is presumed to arise following antigenic stimulation of normal T-cells. 2. Upon oligoclonal expansion of antigen reactive T-cells, somatic mutations are acquired in genes regulating key T-cell survival pathways as well as epigenetic regulators. 3. The leukemic expansion is characterized by clonal T-cell receptor rearrangements, somatic variants (especially somatic activating mutations in the *STAT3* gene), and an activated cytotoxic T-cell phenotype with secretion of inflammatory cytokines and chemokines, such as sFasL. 4. Leukemic LGLs are resistant to Fas-induced apoptosis and are characterized by activated cell survival pathways. Cytopenias, especially neutropenia and anemia, are a common disease feature and the main indicators for treatment. Leukemic LGLs also invade spleen, marrow and other organs where they contribute to cytopenias and autoimmune diseases. Created with BioRender.com.

T-LGL leukemia pathogenesis is likely initiated by antigenic stimulation of cytotoxic T-cell expansion followed by somatic mutational events that activate survival pathways, subvert activation induced cell death, and drive clonal expansion (summarized in **Figure 1**). An abundance of reported genetic modifications and signaling changes point to a reliance on inflammatory and JAK/STAT signaling in LGL leukemia. In fact, nearly all patients show an increase in STAT3 activation (7–9), suggesting a stimulatory role for cytokine signaling pathways. The JAK/STAT signaling cascade is first initiated by cytokines such as IL-6, IL-2, and IL-15 and following activation, leads to transcription of STAT responsive genes that impact survival, proliferation, and immune activation (10).

Furthermore, STAT3 somatic activating mutations are the hallmark genetic lesion of LGL leukemia. Mutations were initially reported in roughly 30-40% of patients (9, 11). The majority of mutations occur in the SH2 domain, the region that mediates dimerization and activation of the STAT3 protein. However, recent publications report mutations in additional regions of the protein, such as the coiled-coil domain, some of which exhibit an activating phenotype. Their inclusion yields an overall *STAT3* somatic mutation rate of >50% in LGL leukemia (12–14).

Cytopenias (neutropenia, anemia, and more rarely thrombocytopenia), splenomegaly, and concomitant autoimmune diseases are the most common clinical manifestations. One of the most common symptoms of LGL leukemia is neutropenia. It is a major health concern, putting patients at risk for infection, pneumonia, or sepsis (11), especially in those with severe neutropenia (<0.5 ×10^9^/L) (15). Numbers vary between cohorts, but as high as 80% of symptomatic patients suffer from a neutrophil count lower than 1.5 × 10^9^/L (16). Immune phenotype also correlates with neutropenia, which is found almost exclusively in CD8+ LGL leukemia (5). In one report, T-LGL leukemia patients with a CD8+, CD3+, CD16+, CD56- phenotype were the most likely to suffer from neutropenia (17). There have been several mechanisms proposed to explain LGL leukemia symptomology including: 1) LGL-secreted humoral factors, 2) LGL bone marrow infiltration, and 3) LGL-mediated cytotoxicity (17). Mechanistic drivers of neutropenia are discussed in more detail in later sections.

## Rheumatoid Arthritis (RA) Association With LGL Leukemia

LGL leukemia is often associated with autoimmune disorders including pure red cell aplasia, celiac disease, and others, but is most commonly associated with rheumatoid arthritis (RA) (18–20). LGL leukemia was first identified as a clonal disorder in 1985 (21). There were several descriptions of a few patients having RA with LGL leukemia around this time; indeed one of the patients in the original description of LGL leukemia was thought to have Felty syndrome, which is characterized by RA, neutropenia, and splenomegaly (22–24). RA is a systemic autoimmune disease characterized by chronic inflammation of the synovial joints, leading to pain, swelling, and destruction of the bone and cartilage (25). RA most commonly becomes symptomatic around 45–60 years of age, and women are two- to threefold more likely to develop RA than men (26). As a standalone clinical entity, RA occurs in ~1% of the world-wide population. However, reports place the incidence of RA in LGL leukemia patients as high as 36% (4, 18, 27). Of note, it is much more commonly observed in patients with T-LGL leukemia compared to those with NK-LGL leukemia (18). In the majority of patients who manifest both T-LGL leukemia and RA, the RA is diagnosed first. In a study of 56 patients with concurrent T-LGL leukemia and RA from a single clinical center, the median time that patients had RA prior to T-LGL leukemia diagnosis was six years, with a range of 0-36 years (28). LGL leukemia is rare in juvenile idiopathic arthritis (JIA) (29), likely because JIA and RA are different pathogenic entities, and has not be reported to have a relationship with late onset RA.

Importantly, once a patient with RA is found to have LGL leukemia, the patient is no longer classified as having RA. Instead, the diagnosis and treatment are centered around the LGL leukemia and the most serious complications associated with the disease (i.e. neutropenia and anemia). In this situation, the RA is considered associated with the LGL leukemia, rather than a separate disease entity. There are no case series comparing arthritis severity in canonical RA and LGL leukemia-associated RA. However, based on case reports, the severity of the arthritis in LGL leukemia appears to be similar to that occurring in canonical RA. The joint damage in both diseases is heterogeneous, with some individuals experiencing mild symptoms, while others have severe erosive joint disease.

Systematic evaluation of the clinical, genetic, and immunologic parallels between LGL leukemia and RA may reveal common mechanisms responsible for the co-occurrence of these two disorders.

## Parallels and Comparisons Between T-LGL Leukemia and RA

**Figure 2:**
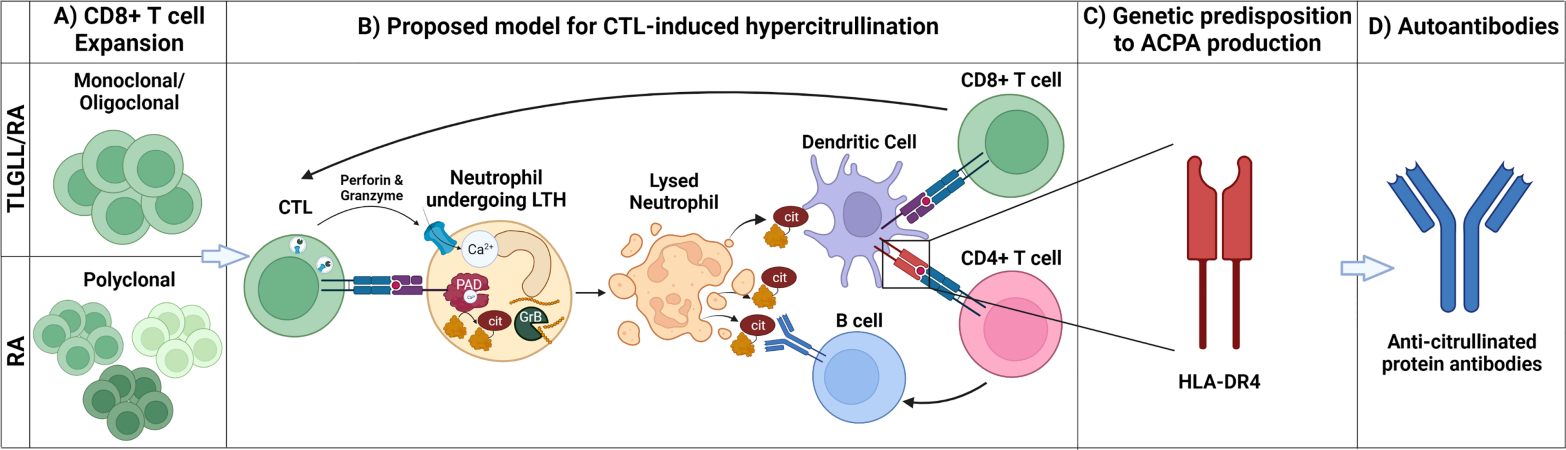
Mechanistic parallels between T-LGL leukemia/RA and canonical RA. **(A)** CD8+ T cell expansion: T-LGL leukemia-associated RA (T-LGLL/RA) and canonical RA (RA) are characterized by the expansion of CD8+ T cells. The CD8+ T cell expansion is oligoclonal/monoclonal in T-LGLL/RA, whereas it is polyclonal in canonical RA. **(B)** Proposed model for CTL-induced hypercitrullination: In this model, clonally expanded CD8+ T cells (CTLs) targeting neutrophils release cytotoxic granules containing perforin and granzymes, inducing leukotoxic hypercitrullination (LTH). Perforin forms pores in the neutrophil membrane, allowing for calcium (Ca^2+^) influx and activation of intracellular PAD enzymes, inducing neutrophil hypercitrullination. In parallel, granzyme B (GrB) cleavage of neutrophil antigens creates neoepitopes. As a result of the disrupted cell membrane, the neutrophils lyse, releasing autoantigens, including citrullinated and GrB-cleaved proteins. Dendritic cells (DCs) engulf these antigens and present them both to CD8+ and CD4+ T cells. The stimulated CD8+ T cells clonally expand and drive a feedforward cycle of neutrophil damage. Stimulated CD4+ T cells provide B cell help, giving rise to antibody-secreting cells producing anti-citrullinated protein antibodies (ACPAs). **(C)** Genetic predisposition to ACPA production: ACPA production is facilitated by the presentation of citrullinated antigens *via* HLA-DRs (e.g., HLA-DR4) encoded by RA-associated HLA-DRB1 susceptibility alleles. The requirement of specific RA-associated HLA-DRs for ACPA production likely explains why, despite having CTL expansion and neutrophil lysis, only a subset of patients with LGL leukemia develop RA. **(D)** Autoantibodies: Circulating APCAs are found in patients with T-LGLL/RA and canonical RA providing a serological record of the breach of immunologic tolerance to citrullinated antigens in both diseases. Created with BioRender.com.

Despite the striking association between T-LGL leukemia and RA, the underlying mechanisms connecting the two disorders remains unknown. There are numerous points of similarity between the RA that develops in the presence and absence of LGL leukemia including common genetic, serologic, and cellular features. These features are discussed below and summarized in **Figure 2**.

### Cytotoxic T-Cells (CTLs) in LGL Leukemia and RA

LGLs themselves are characterized by their large size, azurophilic cytoplasmic granules, low nuclear to cytoplasmic ratio, and round nucleus. In healthy populations, LGLs make up about 10-15% of peripheral blood mononuclear cells (PBMCs), but patients with LGL leukemia can have levels as high as 2- to 40-fold greater than their baseline (27). Diagnosis is supported by increased cell counts of > 2×10^9^/L or lower counts (0.4 – 2×10^9^/L) when the cells are clonal and the disease is paired with the appropriate clinical features such as RA and hematological parameters like cytopenias. Clonality assessment based upon T-cell receptor (TCR) rearrangement in αβ and γδ TCR genes is used to confirm diagnosis if the appropriate cell expansions are observed. Histologically, bone marrow (BM) samples show interstitial infiltrations of linear arrays of cytotoxic cells expressing CD8, cytotoxic granules containing perforin and granzyme B, and/or TIA-1 (30).

The T-LGL leukemia phenotype is typically CD3+, TCRαβ+, CD8+, CD16+, CD45RA+, and CD57+, and cells are CD4−, CD5dim, CD27−, CD28−, CD45RO−. Leukemic CD3+, CD8+ LGLs frequently exhibit relatively equal proportions of CD57- and CD57+ cells, which are proposed to represent progenitor and mature populations, respectively (31, 32). At the phenotypic and transcriptional level, these cells resemble chronically stimulated terminally differentiated cytotoxic T lymphocytes (CTLs), such as those found in the setting of viral infection (33). Additionally, granzymes A, B, H, and K have been shown to be upregulated in LGL leukemia (34). The re-expression of CD45RA, as is observed on T-LGLs, is a feature of a sub population of effector CD8s referred to as “T effector memory cells re-expressing CD45RA” (TEMRA) cells (35). While this suggests that leukemic T-LGLs may derive from TEMRA cells (36), further comparisons using single cell approaches are needed to precisely define this relationship.

Clonal CD8+ T cell expansions have also been observed in the blood of RA patients, in the absence of known T-LGL leukemia, more frequently than in healthy controls (45% vs. 25%, respectively) (37), suggesting that antigen-driven expansion of clonal CTL populations is occurring in RA. In fact, examination of a large cohort of over 500 RA patients revealed clonal expansions in 3.6% of patients. Only 42% of patients with clonal expansions had counts above the threshold of 500 cells/µL typically considered for initial diagnosis of LGL leukemia (38). However, most patients with these clonal T-cell populations had previously been exposed to antirheumatic immunosuppressive treatments (also common treatments for LGL leukemia), which may blunt the progression along a potential continuum between RA and LGL leukemia. Given that over a million people in the US suffer from RA, these findings suggest that clonal T-cell populations are more common than the currently documented incidence of T-LGL leukemia.

As in T-LGL leukemia, the CTLs found in the synovium of RA patients are classified as effector memory or TEMRA cells (39). These cells are clonally expanded and express CD80, CD86, PD-1, and Ki67, indicating an activated and chronically stimulated phenotype (39, 40). They can persist in the joint for years, and CD3+ CD57+ cells accumulate with disease duration (41, 42). Moreover, similar to T-LGL leukemia, synovial CTLs in RA express perforin and granzymes (43). Indeed, an active role of degranulating CTLs in RA pathogenesis is supported by the findings that granzymes A, B and M are elevated in RA synovial fluid (44, 45), and serum levels of granzyme B correlate with disease activity and joint erosion (46). The accumulation of antigen-experienced clonally expanded CTLs in the RA synovium and evidence of active degranulation, implicates these cells in the pathogenesis of RA, but their precise role remains undefined.

### Somatic Mutations in T-LGL Leukemia and RA

STAT3 mutations are the predominant somatic variants in T-LGL leukemia and have been associated with a variety of clinical markers of disease pathogenesis and outcome. A 2019 retrospective study of one of the largest LGL leukemia cohorts to date revealed that STAT3 mutations were associated with low hemoglobin and lower overall survival, as well as severe neutropenia (47). Another recent study confirmed higher rates of neutropenia, severe neutropenia, and cases requiring treatment in STAT3 mutated samples (48). STAT3 mutations are generally found almost exclusively in CD8+ rather than CD4+ patients (5), and more specifically, CD8+ CD16+ CD56- T-LGL leukemia patients exhibit more STAT3 mutations (49).

Numerous studies have associated STAT3 mutations with moderate and severe neutropenia in LGL leukemia (5, 9, 14, 48, 50, 51). STAT3 is a driver of soluble Fas ligand (sFasL) expression in LGLs (52), and sFasL is present at high levels in LGL leukemia patient serum (53). LGLs are resistant to FasL-induced apoptosis due to widespread activation of a network of survival signals (54). However, patient serum is sufficient to activate cell death in normal neutrophils *in vitro* (**Figure 1**). A blocking anti-Fas monoclonal antibody rescued neutrophils from this fate (53). In addition, LGL patients with neutropenia have higher sFasL levels when compared to either healthy donor serum or serum from LGL leukemia patients with normal neutrophil counts. Furthermore, successful treatment has been associated with lower levels of sFasL (17), with methotrexate specifically inducing lower sFasL, and relapsed patients exhibiting increased sFasL (53). Thus, several lines of evidence implicate sFasL as a humoral mediator of neutropenia in LGL leukemia. Further discussion of direct LGL cytotoxic effects on neutrophils is presented below.

Interestingly, T-LGL leukemia patients with STAT3 mutations are more likely to have RA than those without (9, 50, 55–58). Whole exome sequencing in a large T-LGL leukemia cohort identified additional genes with recurrent somatic variants as well as frequent co-mutations of chromatin modifying genes in *STAT3*-mutant T-LGLs (14). Further studies are needed to define additional molecular events that correlate with RA co-occurrence in LGL leukemia.

Recent efforts identified 30 somatic mutations in clonally expanded CTLs of a small cohort of RA patients who did not have a diagnosis of T-LGL leukemia (40). Using a combination of gene targeted and exome sequencing approaches, mutations were identified in immune-related genes, proliferation-associated genes, as well as in other genes (40). Notably, these mutations were all found in clonally expanded CD8+ effector memory T cell populations, suggesting that CD8+ T cells that acquire these somatic mutations may clonally expand and play a pathogenic role in RA. However, it is important to note that somatic mutations were only found in 5/25 patients studied, and most mutations were only found in a single patient. While these data are intriguing, further studies on larger cohorts are needed to identify whether CTL mutations in RA are causal or an effect of the disease and to draw any meaningful parallels between the mutational CTL landscapes in RA and T-LGL leukemia.

### Sex Bias

Although LGL leukemia generally occurs equally in males and females, with some studies showing a slightly increased incidence in males (2), the development of RA in patients with T-LGL leukemia is highly skewed toward females. One study of 56 patients with T-LGL leukemia and RA found that 73% were female (28). This parallels what has been observed in canonical RA for decades, a 3:1 female:male ratio (59, 60). While much more needs to be learned about the mechanism behind this sex bias, the increased risk of RA development in females with T-LGL leukemia suggests parallel mechanisms with canonical RA.

### Immunogenetic Associations

RA is associated with a specific group of *HLA-DRB1* alleles termed the “shared epitope” alleles, so named due to the presence of a common amino acid motif (QKRAA) in the peptide binding groove of the encoded protein (61). The *HLA-DRB1* gene encodes the HLA-DRβ chain of the MHC class II molecule, HLA-DR, which serve as scaffolds for antigen presenting cells to display exogenously derived peptide antigens to CD4+ T helper cells. The HLA-DRB1 locus is highly polymorphic in humans and confers the highest genetic risk for RA development (62). While the risk for RA was initially attributed to HLA-DRB1*04 allelic variants (63), it was later appreciated that a larger group of alleles encoding for the “shared epitope” are collectively associated with RA (61). The most common RA-associated shared epitope alleles include HLA-DRB1*01:01, 01:02, 04:01, 04:04, 04:05, 10:01, and 14:02 (64).

Patients with concurrent T-LGL leukemia and RA are also enriched in HLA-DRB1*04 alleles associated with RA (65, 66). One study showed that 9/10 patients (90%) with T-LGL leukemia and RA expressed HLA-DRB1*04, whereas only 4/12 (33%) of patients with T-LGL leukemia alone expressed HLA-DRB1*04 (66). Two important caveats of these studies are that only HLA-DRB1*04 was evaluated, not other shared epitope alleles, and that individual allelic variants of HLA-DRB1*04 were not considered. This is important since some HLA-DRB1*04 variants are associated with RA (i.e. HLA-DRB1*04:01, 04:04, and 04:05), while others have been found to be protective against RA development and severity (i.e. HLA-DRB1*04:02). Although additional studies are needed to precisely compare the immunogenetic similarities between T-LGL leukemia and RA, the enrichment of RA-associated HLA-DRB1*04 alleles in patients with T-LGL leukemia who develop RA suggests the presence of a shared immunogenetic scaffold.

### Antigen Specificity

Despite the observed clonal expansion and antigen-experienced phenotype, the antigen-specificity of the clonally expanded TEMRA cells in T-LGL leukemia and canonical RA remains largely unknown. One study observed close contact between LGL cells and dendritic cells (DCs) in bone marrow biopsies from patients with LGL leukemia (67). In *ex vivo* experiments, LGLs could be stimulated to proliferate when cultured with autologous bone marrow-derived, but not peripheral blood-derived, DCs, suggesting that these cells are actively responding to an antigen present in the bone marrow microenvironment. More recently, seroreactivity to human T-cell leukemia virus (HTLV-1/2) and human immunodeficiency virus (HIV-1) retroviral epitopes was identified in a subset of LGL leukemia as well as the clinically normal family members of reactive patients (68). There was no evidence of retroviral infection in reactive patients. While this viral seroreactivity has been identified in a subset of LGL leukemia, no unifying antigenic driver has been identified, and this represents a key knowledge gap in the disease.

In RA, one study has shown that RA patients have a population of CTLs that are autoreactive against epitopes from apoptotic cells that are cross-presented by dendritic cells, termed “apoptotic epitopes.” These epitopes include those from vimentin and actin (69). This is interesting given that citrullinated vimentin and actin are both known targets of anti-citrullinated protein antibodies (ACPAs) in patients with RA (70, 71). In RA patients that do not respond to anti-TNF therapy, these CTLs display a TEMRA phenotype and are able to kill Tregs *in vitro* after stimulation with apoptotic epitopes, *via* a NKG2D-dependent mechanism. In addition, immunofluorescence imaging of the synovium of these patients has shown that CTLs interact with Tregs, some of which express cleaved caspase-3, suggesting that these CTLs can kill Tregs *in vivo* (72). Much is still unknown about the epitopes recognized by CTLs in T-LGL leukemia and canonical RA. The definition of the target cells and antigens in these diseases is critical for understanding disease pathogenesis.

### Serologic Profile

A hallmark feature of canonical RA is the formation of high titer autoantibodies targeting a defined set of self-proteins, making them powerful diagnostic biomarkers (73). There are two main autoantibodies that are analyzed clinically: 1) autoantibodies recognizing the Fc-portion of IgG, termed rheumatoid factor (RF); and 2) autoantibodies targeting proteins containing the post translational modification citrulline, termed anti-citrullinated protein antibodies (ACPAs). Each antibody specificity is present in approximately 70% of patients with RA and can co-occur in the same patient as well as exist separately (74). While both RF and ACPAs have high sensitivity for a diagnosis of RA, ACPAs are more specific, suggesting dysregulated protein citrullination and a breach of tolerance to these antigens as key processes in RA. ACPAs are a collection of antibodies targeting a diverse set of proteins in which arginine residues have been post-translationally deiminated by the peptidylarginine deiminase (PAD) enzymes, generating the non-classical amino acid citrulline (75). These antibodies are detected clinically using synthetic cyclic-citrullinated peptides (CCP). In addition, the development of ACPAs is associated with HLA-DRB1 shared epitope alleles (76), implicating this common genetic scaffold in the development of immune responses to citrullinated proteins.

Interestingly, RA-associated autoantibodies are also detected at high levels in individuals with T-LGL leukemia. In a study of 27 patients with T-LGL, 15 (55.6%) were positive for RF, four of whom did not have a diagnosis of RA (77). In a study of 56 T-LGL leukemia and RA cases, 82% were RF positive and 88% were positive for anti-CCP antibodies (28). In a small study comparing ACPA positivity in T-LGL leukemia patients with and without RA, 95% (18/19) of T-LGL leukemia patients with RA had ACPAs, compared to none (0/15) of the patients without RA (78). Importantly, while the data suggest that seropositivity for classic RA autoantibodies may be higher in T-LGL leukemia patients with RA compared to the general RA population, further head-to-head studies are needed to define the serologic overlap between the two disease entities. Together, these data highlight the serological similarity between patients with RA in the presence and absence of T-LGL leukemia, and support the hypothesis that dysregulated protein citrullination is a key pathogenic process both in RA and T-LGL leukemia/RA.

### Treatment

Most patients with LGL leukemia eventually need treatment because of severe or symptomatic neutropenia, anemia, or associated autoimmune conditions. Because LGL leukemia is such a rare disease, most clinical evidence for drug selection is derived from retrospective studies that indicate the efficacy of three main immunosuppressive treatments: methotrexate (MTX), cyclophosphamide, and cyclosporine A (27). Interestingly, these therapies have significant parallels with treatments for canonical RA. MTX is a first-line therapy for RA, and oral cyclophosphamide and cyclosporine A are also useful to control RA (79, 80), although the use of cyclophosphamide is limited because of toxicity and cyclosporine A is reserved for refractory RA. Therefore, LGL leukemia with or without RA is usually treated as a single entity without the need for using additional therapies to treat the concomitant RA, unless joint symptoms persist. Importantly, considering that LGL leukemia is the potential driver of RA in this group of patients, in principle, any treatment controlling the leukemia should be effective in controlling RA.

Similarly, therapies introduced to treat the RA in patients with LGL leukemia have shown benefit in improving hematological parameters associated with the leukemia, including cytopenias and LGL expansion. In particular, rituximab, a monoclonal antibody therapy targeting CD20, has been shown to induce a remarkable 100% hematological response rate (either complete or partial leukemia remission) in small case series and case reports of refractory LGL leukemia with RA (81–84), and in one case of refractory LGL leukemia without RA (85). The JAK3 inhibitor tofacitinib has also been shown to induce hematological improvement in some patients with refractory LGL leukemia and RA (86). The finding that similar therapies are useful in treating both canonical RA and LGL leukemia supports the notion that these diseases share common pathogenic pathways.

### Interrelationship Amongst T-LGL Leukemia, RA and Felty Syndrome

Felty Syndrome (FS) is a rare disorder occurring in 1-3% of RA patients and is defined by the presence of splenomegaly and neutropenia (87). Given its symptomatic overlap with LGL leukemia, there is considerable debate about whether FS and LGL leukemia are distinct or related entities. FS has long been associated with LGL leukemia (88, 89), and LGL leukemia may co-occur in as high as 40% of FS patients (18). Past reports have also observed a high prevalence of HLA-DRB1*04 alleles in both diseases (86.7% in FS; 82.8% in LGL leukemia/RA patients; 31.4% in LGL leukemia patients, which is similar to control population rates) (66) as well as response to methotrexate therapy in both diseases (90). Moreover, FS, LGL leukemia and RA share elevated levels of the cytokines IL-6, HGF, CDCP1 and CXCL10, and the latter correlates with more severe disease activity in RA (91, 92).

Recent studies have applied advanced molecular analyses to further define the relationship between the two diseases. A 2018 analysis of 14 FS patients found that 43% had *STAT3* mutations in the SH2 domain as detected by deep amplicon sequencing. Regardless of mutational status, a majority of bone marrow samples exhibited elevated phospho-STAT3 levels. Many of these patients had a high percentage of lymphocytes, but this did not necessarily equate to overall lymphocytosis. On average, these FS patients had smaller clone sizes than the average T-LGL leukemia patient (91). In 2021, Gorodetskiy et al. stratified FS patients by presence or absence of clonal T cell expansion, classifying those patients with expansions as LGL leukemia/RA (n=56) and the remainder as FS alone (n=25). Interestingly, in contrast to patients with FS, LGL leukemia/RA patients exhibited increased LGL counts >2 x 10e9/L (21% vs. 0% in FS) and *STAT3* mutations (39% vs. 0% in FS) (28). This *STAT3* mutation prevalence in the LGL leukemia/RA group is similar to the frequency in previously published studies in LGL leukemia (9, 93). These data suggest that the extent of clonal T-cell expansion may distinguish LGL leukemia/RA from FS. It remains to be determined if FS patients classified in this manner will later acquire somatic activating mutation in *STAT3* and/or progress to LGL leukemia/RA. LGL leukemia/RA and FS both exhibited CD3+CD8+ T-cells with CD57, CD16 and CD5^-/dim^ expression (28). Notably, T-cell clonality and *STAT3* mutations were detected more frequently in spleen samples than peripheral blood or bone marrow from ten atypical LGL leukemia/RA patients with lymphopenia, severe neutropenia, and marked splenomegaly, emphasizing the potential for LGL leukemia misdiagnosis as FS (94).

Further studies are needed to refine the diagnostic criteria to distinguish between LGL leukemia and FS, if they are indeed distinct diseases. However, substantial challenges remain to the routine application of sensitive molecular methods to uncommon specimens such as bone marrow and spleen material. Increased utilization of T-cell clonality and *STAT3* mutational profiling may lead to increased diagnosis of LGL leukemia within RA and FS patient populations, yet these events are likely detectable in all three diseases with ultrasensitive detection methods.

In summary, canonical RA and the subset of patients with LGL leukemia and RA exhibit an abundance of shared and overlapping demographic, immunologic, serologic, and genetic features. These parallels are unlikely to be fortuitous but evoke a common mechanism for RA development. The following section provides some considerations to explain the connection between these two diseases.

## Proposed Mechanisms for the Relationship Between T-LGL Leukemia and RA

Different models have been proposed for the co-occurrence of T-LGL leukemia and RA. Since RA is generally documented several years before LGL leukemia is diagnosed, it has been questioned whether T-LGL leukemia is a consequence of long-standing RA, whether the leukemia develops as a consequence of RA treatment (38), or whether the clonal expansion of pathogenic CTLs is indeed the driver of RA in these patients. Evidence for these three options will be discussed in detail below, and it is important to note that there may be no single model that can explain all cases of RA occurring in the setting of T-LGL leukemia. Understanding the mechanistic relationship between RA and T-LGL leukemia is critical for understanding disease pathogenesis and identifying effective preventive and treatment strategies for both disorders.

### LGL Leukemia as a Consequence of RA

Clonal CD8+ T cell expansions have been observed in RA, which is not surprising given the chronic autoantigen driven nature of this disease. One possibility for the co-occurrence of RA and T-LGL leukemia is that the clonal expansion of CD8+ T cells in RA may result in the acquisition of *STAT3* and other somatic mutations, T cell transformation, and the development of leukemia. While more frequent clonal CD8+ T cell expansions have been observed in RA compared to healthy controls (45% vs. 25%, respectively), the same study found that the two groups had a similar degree of clonality, and some individuals in both the RA and healthy control groups exhibited expansions comprising ~40% of their CD8+ T cell pool (37). This suggests that although CD8+ T cell expansions are common in RA, they alone cannot explain the concomitant development of RA and LGL leukemia. In addition, T-LGL leukemia can occur in the absence of RA, demonstrating that RA is not a prerequisite for the development of leukemic T-LGLs. Thus, while it may be tempting to speculate that RA is the driver of T-LGL leukemia based on the frequent diagnosis of RA before T-LGL leukemia, it is equally likely that occult low frequency LGL clones initiate the breach of immune tolerance to self-antigens prior to the development of neutropenia and clinical discovery of T-LGL leukemia (see “Pathogenic CTLs as the driver of RA” section).

### LGL Leukemia as a Consequence of RA Treatment

Another possible explanation for the co-occurrence of LGL leukemia and RA is that LGL leukemia develops as a result of the immunomodulating therapies used to treat RA, namely treatment with tumor necrosis factor (TNF) inhibitors. In one study, clonal expansions of LGL cells expressing CD3, CD56, and γδ TCRs were observed in 3.6% (19/529) of RA patients and were found to positively correlate with exposure time to TNF blocking agents (38). However, it is important to note that this phenomenon is not unique to RA. Similar clonal expansions of LGL cells with γδ TCRs have been observed in association with TNF inhibitor use in patients with ankylosing spondylitis (SpA) and psoriatic arthritis (PsA) (95). In addition, a relationship between anti-TNF use for the treatment of irritable bowel disease and the development of hepatosplenic T-cell lymphoma (HSTCL) (96), has been suggested by a literature review study that found 11% (22/200) of HSTCL cases reported in the literature were associated with IBD treatment (97). It remains to be determined if such LGL cell clonal expansions are associated with progression to LGL leukemia in any of the individuals in whom they were detected, and whether treatment may drive or expand an existing pathogenic LGL pool present in these patients. Regardless of the mechanism for their development, the lack of specificity of these clonally expanded LGL cells for RA or LGL leukemia suggests that anti-TNF inhibitor therapy is not likely to be the mechanistic link between RA and T-LGL leukemia.

### LGL Leukemia as the Driver of RA

While not all factors contributing to RA development are known, accumulating evidence suggests a central role for CTLs in RA pathogenesis, both as effectors perpetuating tissue damage and as generators of RA autoantigens (**Figure 2**). This latter role may be the key to linking T-LGL leukemia to RA development. We postulate that, in people with T-LGL leukemia and concomitant RA, the resulting autoimmunity represents a paraneoplastic syndrome caused by the expanded T-LGL clones. Moreover, parallel CTL-driven mechanisms may contribute to the development of RA in people without T-LGL leukemia.

This hypothesis is supported by the finding that a subset of RA patients have evidence of killer cell pathway activation in their joints in association with a form of lytic neutrophil cell death, termed leukotoxic hypercitrullination (LTH) (98, 99). LTH has been found to be unique among cell death and activation stimuli tested to date in its ability to hyperactivate the intracellular calcium-dependent peptidyl arginine deiminase (PAD) enzymes, leading to widespread protein citrullination in a pattern similar to that found in cells of the RA joint. LTH can be triggered by both host and pathogen-derived pore forming proteins, which allow the influx of extracellular calcium into the cell and hyperactivation of the intracellular PAD enzymes (98–100). In the subset of RA patients with LTH-associated hypercitrullination in the joint, the pore forming protein perforin was identified as the causative factor in the ability of killer cells to induce hypercitrullination in target neutrophils (98). The physiologic role of perforin is to form pores in the membrane of target cells to facilitate the delivery of granzymes, which subsequently cleave intracellular proteins, including caspases, to induce apoptosis *via* the extrinsic pathway. The observation that hypercitrullination was found in synovial fluid cells from a subset of patients with activation of the extrinsic apoptosis pathway, implicates CTL killing of neutrophils in the generation of citrullinated autoantigens in a subset of individuals (98).

A recent study on target cells engineered to express PAD2 or PAD4, two key citrullinating enzymes strongly implicated in RA pathogenesis and highly expressed by neutrophils, demonstrated a combinatorial effect of perforin and granzymes on the creation of autoantigens recognized by sera from RA patients (101). It has been hypothesized that a potential consequence of granzyme-mediated cleavage of self-proteins during the induction of target cell apoptosis is the generation of neoepitopes that may lead to the breach of immunologic tolerance and development of autoimmunity (102). The serine protease granzyme B has been most heavily studied in this regard after it was shown that the majority of autoantigens targeted across the spectrum of systemic autoimmune diseases are substrates for this protease. It was observed that a different pattern of protein fragments was generated when these antigens were cleaved by granzyme B compared to the effector caspase, caspase 8, which has a similar preference for cleaving substrates after aspartic acid residues (103). Together, these studies suggest that CTLs have the potential to modify the autoantigen pool in target cells, both by inducing hypercitrullination in PAD-expressing cells and by granzyme B-mediated cleavage of target cell proteins.

A review of granzyme B-cleaved autoantigens in systemic autoimmunity further revealed that granzyme B cleavage sites and autoreactive B and/or T cell epitopes tend to co-cluster within proteins, suggesting a causal relationship (104). This was demonstrated experimentally for PAD4, which is both a citrullinating enzyme and a target autoantigen in a subset of RA patients with the most destructive joint disease (105–108). In this study, cleavage of PAD4 by granzyme B was found to induce discrete changes in the PAD4 protein structure in regions adjacent to and remote from the granzyme B cleavage site (109). These structural changes were associated with increased presentation of peptide epitopes derived from these regions by an RA-associated HLA-DR allele. Furthermore, the granzyme B-enhanced epitopes were able to stimulate CD4+ T cell responses in patients with RA, suggesting that this process may occur *in vivo*. The findings that citrullination and granzyme B cleavage have the capacity to modify the repertoire of self-proteins present in target cells killed by CTLs coupled with the longstanding observation that RA is present in a subset of patients with T-LGL leukemia, supports the model that T-LGLs are drivers of RA development in individuals with concurrent leukemia and RA.

## Unanswered Questions and Future Research Directions

As detailed above, there are numerous clinical, genetic, and therapeutic overlaps between LGL leukemia and RA (**Figure 2**). It remains to be determined if the clonal CTL expansions detected in a subset of RA patients represent the early stages of a continuum between RA and LGL leukemia. If so, they may represent a biomarker of leukemic risk that warrants increased testing and monitoring. In addition, the cause of the classically observed neutropenia that is prominent in T-LGL leukemia remains unknown, but one hypothesis is the active killing of neutrophils by pathogenic CTL clones. It will be important to determine if direct CTL killing of neutrophils is a uniting feature of both disorders, as it could be responsible for the neutropenia observed in LGL leukemia and be a potent inducer of citrullinated and granzyme B-cleaved autoantigens in both diseases. Future study on the mechanistic parallels between T-LGL leukemia and RA will be critical to elucidate causal pathways and target antigens, in order to develop novel mechanism-guided treatments for these related disorders.

## Author Contributions

KM and KA: writing, figure generation. FA and TL: concept development, critical review. DF and ED: writing, concept development, critical review. All authors contributed to the article and approved the submitted version.

## Author Disclaimer

The content is solely the responsibility of the authors and does not necessarily represent the official views of the National Institutes of Health.

## Conflict of Interest

TL is on the Scientific Advisory Board and has stock options for Keystone Nano, Bioniz Therapeutics and Dren Bio. TL and DF received honoraria from Kymera Therapeutics. DF has research funding from AstraZeneca. ED and FA are coauthors on a licensed patent related to human autoantibodies specific for PAD3 and their use in the diagnosis and treatment of rheumatoid arthritis and related diseases (US patent no. 8,975,033), and are coauthors on a provisional patent related to anti-PAD2 antibody for treating and evaluating rheumatoid arthritis (US patent no. 62/481,158). FA has received consulting fees and/or honoraria from Celgene and Advise Connect Inspire. There are no conflicts of interest with the work presented in this manuscript.

The remaining authors declare that the research was conducted in the absence of any commercial or financial relationships that could be construed as a potential conflict of interest.

## Publisher’s Note

All claims expressed in this article are solely those of the authors and do not necessarily represent those of their affiliated organizations, or those of the publisher, the editors and the reviewers. Any product that may be evaluated in this article, or claim that may be made by its manufacturer, is not guaranteed or endorsed by the publisher.

## References

[1] Moignet A Lamy T. Latest Advances in the Diagnosis and Treatment of Large Granular Lymphocytic Leukemia. Am Soc Clin Oncol Educ book Am Soc Clin Oncol Annu Meet (2018) 616–25. doi: 10.1200/EDBK_200689 30231346

[2] Dinmohamed AG Brink M Visser O Jongen-Lavrencic M. Population-Based Analyses Among 184 Patients Diagnosed With Large Granular Lymphocyte Leukemia in the Netherlands Between 2001 and 2013. Leukemia (2016) 30:1449–51. doi: 10.1038/leu.2016.68 27055870

[3] Shah MV Hook CC Call TG Go RS. A Population-Based Study of Large Granular Lymphocyte Leukemia. Blood Cancer J (2016) 6:e455. doi: 10.1038/bcj.2016.59 27494824 PMC5022177

[4] Lamy T Moignet A Loughran TP. LGL Leukemia: From Pathogenesis to Treatment. Blood (2017) 129:1082–94. doi: 10.1182/blood-2016-08-692590 28115367

[5] Rivero A Mozas P Jiménez L López-guerra M Colomer D Bataller A. Clinicobiological Characteristics and Outcomes of Patients With T-Cell Large Granular Lymphocytic Leukemia and Chronic Lymphoproliferative Disorder of Natural Killer Cells From a Single Institution. Cancers (Basel) (2021) 13:3900. doi: 10.3390/CANCERS13153900/S1 34359799 PMC8345581

[6] Loughran TP Zickl L Olson TL Wang V Zhang D Rajala HLM. Immunosuppressive Therapy of LGL Leukemia: Prospective Multicenter Phase II Study by the Eastern Cooperative Oncology Group (E5998). Leukemia (2015) 29:886–94. doi: 10.1038/leu.2014.298 PMC4377298 25306898

[7] Epling-Burnette PK Liu JH Catlett-Falcone R Turkson J Oshiro M Kothapalli R. Inhibition of STAT3 Signaling Leads to Apoptosis of Leukemic Large Granular Lymphocytes and Decreased Mcl-1 Expression. J Clin Invest (2001) 107:351–62. doi: 10.1172/JCI9940 PMC199188 11160159

[8] Andersson EI Rajala HLM Eldfors S Ellonen P Olson T Jerez A. Novel Somatic Mutations in Large Granular Lymphocytic Leukemia Affecting the STAT-Pathway and T-Cell Activation. Blood Cancer J (2013) 3. doi: 10.1038/bcj.2013.65 PMC3877422 24317090

[9] Koskela HLM Eldfors S Ellonen P van Adrichem AJ Kuusanmäki H Andersson EI. Somatic STAT3 Mutations in Large Granular Lymphocytic Leukemia. N Engl J Med (2012) 366:1905–13. doi: 10.1056/NEJMoa1114885 PMC3693860 22591296

[10] Abroun S Saki N Ahmadvand M Asghari F Salari F Rahim F. STATs: An Old Story, Yet Mesmerizing. Cell J (2015) 17:395–411. doi: 10.22074/cellj.2015.1 26464811 PMC4601860

[11] Gazitt T Loughran TP. Chronic Neutropenia in LGL Leukemia and Rheumatoid Arthritis. Hematology (2017) 2017:181–6. doi: 10.1182/asheducation-2017.1.181 PMC6142558 29222254

[12] Moosic KB Paila U Olson KC Dziewulska K Wang TT Xing JC. Genomics of LGL Leukemia and Select Other Rare Leukemia/Lymphomas. Best Pract Res Clin Haematol (2019) 32:196–206. doi: 10.1016/J.BEHA.2019.06.003 31585620 PMC6779335

[13] Andersson E Kuusanmäki H Bortoluzzi S Lagström S Parsons A Rajala H. Activating Somatic Mutations Outside the SH2-Domain of STAT3 in LGL Leukemia. Leukemia (2016) 30:1204–8. doi: 10.1038/leu.2015.263 PMC4814354 26419508

[14] Cheon H Xing JC Moosic KB Ung J Chan V Chung DS. Genomic Landscape of TCR Alpha-Beta and TCR Gamma-Delta T-Large Granular Lymphocyte Leukemia. Blood (2022). doi: 10.1182/BLOOD.2021013164 PMC9121841 35015834

[15] Sokol L. Large Granular Lymphocyte Leukemia. Oncologist (2006) 11:263–73. doi: 10.1634/theoncologist.11-3-263 16549811

[16] Zhang R Shah MV Loughran TP. The Root of Many Evils: Indolent Large Granular Lymphocyte Leukaemia and Associated Disorders. Hematol Oncol (2010) 28:105–17. doi: 10.1002/hon.917 PMC4377226 19645074

[17] Calabretto G Teramo A Barilà G Vicenzetto C Gasparini VR Semenzato G. Neutropenia and Large Granular Lymphocyte Leukemia: From Pathogenesis to Therapeutic Options. Cells (2021) 10:2800. doi: 10.3390/CELLS10102800 34685780 PMC8534439

[18] Bockorny B Dasanu CA. Autoimmune Manifestations in Large Granular Lymphocyte Leukemia. Clin Lymphoma Myeloma Leuk (2012) 12:400–5. doi: 10.1016/j.clml.2012.06.006 22999943

[19] Cheon HJ Dziewulska KH Moosic KB Olson KC Gru AA Feith DJ. Advances in the Diagnosis and Treatment of Large Granular Lymphocytic Leukemia. Curr Hematol Malig Rep (2020) 15:103–12. doi: 10.1007/S11899-020-00565-6 PMC7234906 32062772

[20] Dziewulska KH Moosic KB Cheon H Olson KC Feith DJ Loughran TP. Wiley (2021) 183–201. doi: 10.1002/9781119671336.CH14

[21] Loughran TP Kadin ME Starkebaum G Abkowitz JL Clark EA Disteche C. Leukemia of Large Granular Lymphocytes: Association With Clonal Chromosomal Abnormalities and Autoimmune Neutropenia, Thrombocytopenia, and Hemolytic Anemia. Ann Intern Med (1985) 102:169–75. doi: 10.7326/0003-4819-102-2-169 3966754

[22] Chan W Check I Schick C Brynes R Kateley J Winton E. A Morphologic and Immunologic Study of the Large Granular Lymphocyte in Neutropenia With T Lymphocytosis. Blood (1984) 63:1133–40. doi: 10.1182/BLOOD.V63.5.1133.1133 6231966

[23] Linch DC Newland AC Tumbull AL Knott LJ MacWhannel A Beverley P. Unusual T Cell Proliferations and Neutropenia in Rheumatoid Arthritis: Comparison With Classical Felty’s Syndrome. Scand J Haematol (1984) 33:342–50. doi: 10.1111/J.1600-0609.1984.TB00705.X 6334354

[24] Wallis WJ Loughran TP Kadin ME Clark EA Starkebaum GA. Polyarthritis and Neutropenia Associated With Circulating Large Granular Lymphocytes. Ann Intern Med (1985) 103:357–62. doi: 10.7326/0003-4819-103-3-357 4026084

[25] Arend WP Firestein GS. Pre-Rheumatoid Arthritis: Predisposition and Transition to Clinical Synovitis. Nat Rev Rheumatol (2012) 8:573–86. doi: 10.1038/NRRHEUM.2012.134 22907289

[26] Rantapää Dahlqvist S Andrade F. Individuals at Risk of Seropositive Rheumatoid Arthritis: The Evolving Story. J Intern Med (2019) 286:627–43. doi: 10.1111/JOIM.12980 PMC6878216 31562671

[27] Lamy T Loughran TP. How I Treat LGL Leukemia. Blood (2011) 117:2764–74. doi: 10.1182/blood-2010-07-296962 PMC3062292 21190991

[28] Gorodetskiy VR Sidorova YV Kupryshina NA Vasilyev VI Probatova NA Ryzhikova NV. Analysis of a Single-Institution Cohort of Patients With Felty’s Syndrome and T-Cell Large Granular Lymphocytic Leukemia in the Setting of Rheumatoid Arthritis. Rheumatol Int (2021) 41:147. doi: 10.1007/S00296-020-04757-4 33280072 PMC7806571

[29] Blanchong CA Olshefski R Kahwash S. Large Granular Lymphocyte Leukemia: Case Report of Chronic Neutropenia and Rheumatoid Arthritis-Like Symptoms in a Child. Pediatr Dev Pathol (2001) 4:94–9. doi: 10.1007/S100240010126 11200497

[30] Morice WG Jevremovic D Hanson CA. The Expression of the Novel Cytotoxic Protein Granzyme M by Large Granular Lymphocytic Leukaemias of Both T-Cell and NK-Cell Lineage: An Unexpected Finding With Implications Regarding the Pathobiology of These Disorders. Br J Haematol (2007) 137:237–9. doi: 10.1111/J.1365-2141.2007.06564.X 17408463

[31] Melenhorst JJ Eniafe R Follmann D Molldrem J Kirby M El Ouriaghli F. Barrett AJ. T-Cell Large Granular Lymphocyte Leukemia Is Characterized by Massive TCRBV-Restricted Clonal CD8 Expansion and a Generalized Overexpression of the Effector Cell Marker CD57. Hematol J Off J Eur Haematol Assoc (2003) 4:18–25. doi: 10.1038/SJ.THJ.6200212 12692516

[32] Melenhorst JJ Sorbara L Kirby M Hensel NF John Barrett A. Large Granular Lymphocyte Leukaemia is Characterized by a Clonal T-Cell Receptor Rearrangement in Both Memory and Effector CD8(+) Lymphocyte Populations. Br J Haematol (2001) 112:189–94. doi: 10.1046/J.1365-2141.2001.02509.X 11167801

[33] Wlodarski MW Nearman Z Jankowska A Babel N Powers J Leahy P. Phenotypic Differences Between Healthy Effector CTL and Leukemic LGL Cells Support the Notion of Antigen-Triggered Clonal Transformation in T-LGL Leukemia. J Leukoc Biol (2008) 83:589–601. doi: 10.1189/JLB.0107073 18086899 PMC2629660

[34] Kothapalli R Nyland SB Kusmartseva I Bailey RD McKeown TM Loughran TP. Constitutive Production of Proinflammatory Cytokines RANTES, MIP-1beta and IL-18 Characterizes LGL Leukemia. Int J Oncol (2005) 26:529–35. doi: 10.3892/IJO.26.2.529/HTML 15645140

[35] Verma K Ogonek J Varanasi PR Luther S Bünting I Thomay K. Human CD8+ CD57- TEMRA Cells: Too Young to be Called “Old”. PloS One (2017) 12:e0177405. doi: 10.1371/JOURNAL.PONE.0177405 28481945 PMC5421808

[36] Yang J Epling-Burnette PK Painter JS Zou J Bai F Wei S. Antigen Activation and Impaired Fas-Induced Death-Inducing Signaling Complex Formation in T-Large-Granular Lymphocyte Leukemia. Blood (2008) 111:1610–6. doi: 10.1182/blood-2007-06-093823 PMC2214759 17993614

[37] Fitzgerald JE Ricalton NS Meyer AC West SG Kaplan H Behrendt C. Analysis of Clonal CD8+ T Cell Expansions in Normal Individuals and Patients With Rheumatoid Arthritis. J Immunol (1995) 154:3538–47. 7897233

[38] Schwaneck EC Renner R Junker L Einsele H Gadeholt O Geissinger E. Prevalence and Characteristics of Persistent Clonal T Cell Large Granular Lymphocyte Expansions in Rheumatoid Arthritis. Arthritis Rheumatol (2018) 70:1914–22. doi: 10.1002/art.40654 29938921

[39] Cho BA Sim JH Park JA Kim HW Yoo WH Lee SH. Characterization of Effector Memory CD8+ T Cells in the Synovial Fluid of Rheumatoid Arthritis. J Clin Immunol (2012) 32:709–20. doi: 10.1007/S10875-012-9674-3 22367266

[40] Savola P Kelkka T Rajala HL Kuuliala A Kuuliala K Eldfors S. Somatic Mutations in Clonally Expanded Cytotoxic T Lymphocytes in Patients With Newly Diagnosed Rheumatoid Arthritis. Nat Commun (2017) 8:15869. doi: 10.1038/NCOMMS15869 28635960 PMC5482061

[41] Masuko-Hongo K Sekine T Ueda S Kobata T Yamamoto K Nishioka K. Long-Term Persistent Accumulation of CD8+ T Cells in Synovial Fluid of Rheumatoid Arthritis. Ann Rheum Dis (1997) 56:613–21. doi: 10.1136/ARD.56.10.613 PMC1752266 9389223

[42] D’Angeac AD Monier S Jorgensen C Gao Q Travaglio-Encinoza A Bologna C. Increased Percentage of CD3+, CD57+ Lymphocytes in Patients With Rheumatoid Arthritis. Correlation With Duration of Disease. Arthritis Rheum (1993) 36:608–12. doi: 10.1002/ART.1780360506 7683880

[43] Zhang F Wei K Slowikowski K Fonseka CY Rao DA Kelly S. Defining Inflammatory Cell States in Rheumatoid Arthritis Joint Synovial Tissues by Integrating Single-Cell Transcriptomics and Mass Cytometry. Nat Immunol (2019) 20:928–42. doi: 10.1038/S41590-019-0378-1 PMC6602051 31061532

[44] Tak PP Spaeny-Dekking L Kraan MC Breedveld FC Froelich CJ Hack CE. The Levels of Soluble Granzyme A and B are Elevated in Plasma and Synovial Fluid of Patients With Rheumatoid Arthritis (RA). Clin Exp Immunol (1999) 116:366–70. doi: 10.1046/J.1365-2249.1999.00881.X PMC1905268 10337032

[45] Shan L van den Hoogen LL Meeldijk J Kok HM Jongeneel LH Boes M. Increased Intra-Articular Granzyme M May Trigger Local IFN-λ1/IL-29 Response in Rheumatoid Arthritis. Clin Exp Rheumatol (2020) 38:220–6. 31172927

[46] Qiao J Zhou M Li Z Ren J Gao G Zhen J. Elevated Serum Granzyme B Levels are Associated With Disease Activity and Joint Damage in Patients With Rheumatoid Arthritis. J Int Med Res (2020) 48:300060520962954. doi: 10.1177/0300060520962954 33143503 PMC7780569

[47] Barilà G Teramo A Calabretto G Vicenzetto C Gasparini VR Pavan L. Stat3 Mutations Impact on Overall Survival in Large Granular Lymphocyte Leukemia: A Single-Center Experience of 205 Patients. Leuk 2019 344 (2019) 34:1116–24. doi: 10.1038/s41375-019-0644-0 31740810

[48] Muñoz-García N Jara-Acevedo M Caldas C Bárcena P López A Puig N. STAT3 and STAT5B Mutations in T/NK-Cell Chronic Lymphoproliferative Disorders of Large Granular Lymphocytes (LGL): Association With Disease Features. Cancers (Basel) (2020) 12:1–20. doi: 10.3390/CANCERS12123508 PMC7760806 33255665

[49] Teramo A Barila G Calabretto G Ercolin C Lamy T Moignet A. STAT3 Mutation Impacts Biological and Clinical Features of T-LGL Leukemia. Oncotarget (2017) 8:61876–89. doi: 10.18632/ONCOTARGET.18711 PMC5617471 28977911

[50] Jerez A Clemente MJ Makishima H Koskela H LeBlanc F Ng KP. STAT3 Mutations Unify the Pathogenesis of Chronic Lymphoproliferative Disorders of NK Cells and T-Cell Large Granular Lymphocyte Leukemia. Blood (2012) 120:3048. doi: 10.1182/BLOOD-2012-06-435297 22859607 PMC3471515

[51] Barilà G Calabretto G Teramo A Vicenzetto C Gasparini VR Semenzato G. T Cell Large Granular Lymphocyte Leukemia and Chronic NK Lymphocytosis. Best Pract Res Clin Haematol (2019) 32:207–16. doi: 10.1016/J.BEHA.2019.06.006 31585621

[52] Teramo A Barilà G Calabretto G Ercolin C Lamy T Moignet A. STAT3 Mutation Impacts Biological and Clinical Features of T-LGL Leukemia . Oncotarget (2017) 8:61876–89. doi: 10.18632/oncotarget.18711 PMC5617471 28977911

[53] Liu JH Wei S Lamy T Epling-Burnette PK Starkebaum G Djeu JY. Chronic Neutropenia Mediated by Fas Ligand (2000). Blood 95:3219–22. 10807792

[54] Zhang R Shah MV Yang J Nyland SB Liu X Yun JK. Network Model of Survival Signaling in Large Granular Lymphocyte Leukemia. Proc Natl Acad Sci USA (2008) 105:16308–13. doi: 10.1073/PNAS.0806447105 PMC2571012 18852469

[55] Sanikommu SR Clemente MJ Chomczynski P Afable MG Jerez A Thota S. Clinical Features and Treatment Outcomes in Large Granular Lymphocytic Leukemia (LGLL). Leuk Lymphoma (2018) 59:416–22. doi: 10.1080/10428194.2017.1339880 PMC8694069 28633612

[56] Rajala HLM Olson T Clemente MJ Lagström S Ellonen P Lundan T. The Analysis of Clonal Diversity and Therapy Responses Using STAT3 Mutations as a Molecular Marker in Large Granular Lymphocytic Leukemia. Haematologica (2015) 100:91. doi: 10.3324/HAEMATOL.2014.113142 25281507 PMC4281318

[57] Shi M He R Feldman AL Viswanatha DS Jevremovic D Chen D. TAT3 Mutation and Its Clinical and Histopathologic Correlation in T-Cell Large Granular Lymphocytic Leukemia. Hum Pathol (2018) 73:74–81. doi: 10.1016/J.HUMPATH.2017.12.014 29288042

[58] Naji Rad S Rafiee B Raju G Solhjoo M Anand P. T-Cell Large Granular Lymphocyte Leukemia in a Patient With Rheumatoid Arthritis. J Investig Med High Impact Case Rep (2020) 8:2324709620941303. doi: 10.1177/2324709620941303 PMC7357018 32646239

[59] van Vollenhoven RF. Sex Differences in Rheumatoid Arthritis: More Than Meets the Eye. BMC Med (2009) 7:12. doi: 10.1186/1741-7015-7-12 19331649 PMC2670321

[60] Linos A Worthington JW O’fallon M Kurland LT. The Epidemiology of Rheumatoid Arthritis in Rochester, Minnesota: A Study of Incidence, Prevalence, and Mortality. Am J Epidemiol (1980) 111:87–98. doi: 10.1093/OXFORDJOURNALS.AJE.A112878 7352462

[61] Gregersen PK Silver J Winchester RJ. The Shared Epitope Hypothesis. An Approach to Understanding the Molecular Genetics of Susceptibility to Rheumatoid Arthritis. Arthritis Rheum (1987) 30:1205–13. doi: 10.1002/ART.1780301102 2446635

[62] Burton PR Clayton DG Cardon LR Craddock N Deloukas P Duncanson A. Genome-Wide Association Study of 14,000 Cases of Seven Common Diseases and 3,000 Shared Controls. Nature (2007) 447:661–78. doi: 10.1038/NATURE05911 PMC2719288 17554300

[63] Stastny P. Association of the B-Cell Alloantigen DRw4 With Rheumatoid Arthritis. N Engl J Med (1978) 298:869–71. doi: 10.1056/NEJM197804202981602 147420

[64] Holoshitz J. The Rheumatoid Arthritis HLA-DRB1 Shared Epitope. Curr Opin Rheumatol (2010) 22:293–8. doi: 10.1097/BOR.0B013E328336BA63 PMC2921962 20061955

[65] Coakley G Brooks D Iqbal M Kondeatis E Vaughan R Loughran TP. Major Histocompatility Complex Haplotypic Associations in Felty’s Syndrome and Large Granular Lymphocyte Syndrome Are Secondary to Allelic Association With HLA-DRB1 *0401. Rheumatol (Oxford) (2000) 39:393–8. doi: 10.1093/RHEUMATOLOGY/39.4.393 10817772

[66] Starkebaum G Loughran TP Gaur LK Davis P Nepom BS. Immunogenetic Similarities Between Patients With Felty’s Syndrome and Those With Clonal Expansions of Large Granular Lymphocytes in Rheumatoid Arthritis. Arthritis Rheum (1997) 40:624–6. doi: 10.1002/ART.1780400406 9125243

[67] Zambello R Berno T Cannas G Baesso I Binotto G Bonoldi E. Phenotypic and Functional Analyses of Dendritic Cells in Patients With Lymphoproliferative Disease of Granular Lymphocytes (LDGL). Blood (2005) 106:3926–31. doi: 10.1182/BLOOD-2005-05-1972 16091452

[68] Nyland SB Feith DJ Poss M Olson TL Krissinger DJ Poiesz BJ. Retroviral Sero-Reactivity in LGL Leukaemia Patients and Family Members. Br J Haematol (2020) 188:522–7. doi: 10.1111/BJH.16223 PMC7012702 31608437

[69] Citro A Scrivo R Martini H Martire C De Marzio P Vestri AR. CD8+ T Cells Specific to Apoptosis-Associated Antigens Predict the Response to Tumor Necrosis Factor Inhibitor Therapy in Rheumatoid Arthritis. PloS One (2015) 10:e0128607. doi: 10.1371/JOURNAL.PONE.0128607 26061065 PMC4465029

[70] Darrah E Rosen A Giles JT Andrade F. Peptidylarginine Deiminase 2, 3 and 4 Have Distinct Specificities Against Cellular Substrates: Novel Insights Into Autoantigen Selection in Rheumatoid Arthritis. Ann Rheum Dis (2012) 71:92–8. doi: 10.1136/ARD.2011.151712 PMC3302156 21859690

[71] Ménard HA Lapointe E Rochdi MD Zhou ZJ. Insights Into Rheumatoid Arthritis Derived From the Sa Immune System. Arthritis Res (2000) 2:429–32. doi: 10.1186/AR122 PMC128869 11094453

[72] Cammarata I Martire C Citro A Raimondo D Fruci D Melaiu O. Counter-Regulation of Regulatory T Cells by Autoreactive CD8 + T Cells in Rheumatoid Arthritis. J Autoimmun (2019) 99:81–97. doi: 10.1016/J.JAUT.2019.02.001 30777378

[73] Aletaha D Neogi T Silman AJ Funovits J Felson DT Bingham CO. Rheumatoid Arthritis Classification Criteria: An American College of Rheumatology/European League Against Rheumatism Collaborative Initiative. Ann Rheum Dis (2010) 69:1580–8. doi: 10.1136/ARD.2010.138461 20699241

[74] Martinez-Prat L Nissen MJ Lamacchia C Bentow C Cesana L Roux-Lombard P. Comparison of Serological Biomarkers in Rheumatoid Arthritis and Their Combination to Improve Diagnostic Performance. Front Immunol (2018) 9:1113. doi: 10.3389/FIMMU.2018.01113 29928272 PMC5997814

[75] Willemze A Trouw LA Toes REM Huizinga TWJ. The Influence of ACPA Status and Characteristics on the Course of RA. Nat Rev Rheumatol (2012) 8:144–52. doi: 10.1038/NRRHEUM.2011.204 22293763

[76] Huizinga TWJ Amos CI van der Helm-Van Mil AHM Chen W Van Gaalen FA. Refining the Complex Rheumatoid Arthritis Phenotype Based on Specificity of the HLA-DRB1 Shared Epitope for Antibodies to Citrullinated Proteins. Arthritis Rheum (2005) 52:3433–8. doi: 10.1002/ART.21385 16255021

[77] Gentile TC Wener MH Starkebaum G Loughran TP. Humoral Immune Abnormalities in T-Cell Large Granular Lymphocyte Leukemia. Leuk Lymphoma (1996) 23:365–70. doi: 10.3109/10428199609054840 9031118

[78] Different Citrullination Profiles in Spontaneous Versus Leukemia-Associated Rheumatoid Arthritis, in: ACR Meeting Abstracts. Available at: https://acrabstracts.org/abstract/different-citrullination-profiles-in-spontaneous-versus-leukemia-associated-rheumatoid-arthritis/ (Accessed January 27, 2022).

[79] Suarez-Almazor ME Belseck E Shea B Tugwell P Wells GA. Cyclophosphamide for Treating Rheumatoid Arthritis. Cochrane Database Syst Rev (2000) 2010:CD001157. doi: 10.1002/14651858.CD001157 11034702

[80] Kitahara K Kawai S. Cyclosporine and Tacrolimus for the Treatment of Rheumatoid Arthritis. Curr Opin Rheumatol (2007) 19:238–45. doi: 10.1097/BOR.0B013E328099AF80 17414949

[81] Lobbes H Dervout C Toussirot E Felten R Sibilia J Wendling D. Rituximab for Rheumatoid Arthritis-Associated Large Granular Lymphocytic Leukemia, A Retrospective Case Series. Semin Arthritis Rheum (2020) 50:1109–13. doi: 10.1016/J.SEMARTHRIT.2020.05.020 32920324

[82] Cornec D Devauchelle-Pensec V Jousse-Joulin S Marhadour T Ugo V Berthou C. Long-Term Remission of T-Cell Large Granular Lymphocyte Leukemia Associated With Rheumatoid Arthritis After Rituximab Therapy. Blood (2013) 122:1583–6. doi: 10.1182/BLOOD-2013-03-491464 23869084

[83] Raposo A Cerqueira M Costa J Sousa Neves J Teixeira F Afonso C. Rheumatoid Arthritis and Associated Large Granular Lymphocytic Leukemia–Successful Treatment With Rituximab. Acta Reumatol Port (2015) 40(4):384–7. 26922203

[84] Verhoeven F Guillot X Prati C Wendling D. Treatment of Pseudo Felty’s Syndrome: Is There a Place for Rituximab? Jt Bone Spine (2015) 82:196–9. doi: 10.1016/J.JBSPIN.2014.12.001 25623522

[85] Ibrahim U Parylo S Kedia S Hussein S Atallah JP. Large Granular Lymphocytic Leukemia: A Report of Response to Rituximab. Case Rep Hematol (2017) 2017:1–3. doi: 10.1155/2017/7506542 PMC5539931 28804660

[86] Bilori B Thota S Clemente MJ Patel B Jerez A Afable M. Tofacitinib as a Novel Salvage Therapy for Refractory T-Cell Large Granular Lymphocytic Leukemia. Leuk 2015 2912 (2015) 29:2427–9. doi: 10.1038/leu.2015.280 26449659

[87] Balint GP Balint PV. Felty’s Syndrome. Best Pract Res Clin Rheumatol (2004) 18:631–45. doi: 10.1016/J.BERH.2004.05.002 15454123

[88] Bowman SJ Bhavnani M Geddes GC Corrigall V Boylston AW Panayi GS. Large Granular Lymphocyte Expansions in Patients With Felty’s Syndrome: Analysis Using Anti-T Cell Receptor V Beta-Specific Monoclonal Antibodies. Clin Exp Immunol (1995) 101:18–24. doi: 10.1111/J.1365-2249.1995.TB02271.X PMC1553309 7621587

[89] Loughran TP Starkebaum G Kidd P Neiman P. Clonal Proliferation of Large Granular Lymphocytes in Rheumatoid Arthritis. Arthritis Rheum (1988) 31:31–6. doi: 10.1002/ART.1780310105 3345230

[90] Liu X Loughran TP. The Spectrum of LGL and Felty’s Syndrome. Curr Opin Hematol (2011) 18:254–9. doi: 10.1097/MOH.0b013e32834760fb PMC4377227 21546829

[91] Savola P Brück O Olson T Kelkka T Kauppi MJ Kovanen PE. Somatic STAT3 Mutations in Felty Syndrome: An Implication for a Common Pathogenesis With Large Granular Lymphocyte Leukemia. Haematologica (2018) 103:304–12. doi: 10.3324/haematol.2017.175729 PMC5792275 29217783

[92] Pandya JM Lundell AC Andersson K Nordström I Theander E Rudin A. Blood Chemokine Profile in Untreated Early Rheumatoid Arthritis: CXCL10 as a Disease Activity Marker. Arthritis Res Ther (2017) 19:20. doi: 10.1186/S13075-017-1224-1 28148302 PMC5289001

[93] Jerez A Clemente MJ Makishima H Koskela H Leblanc F Peng Ng K. STAT3 Mutations Unify the Pathogenesis of Chronic Lymphoproliferative Disorders of NK Cells and T-Cell Large Granular Lymphocyte Leukemia. Blood (2012) 120:3048–57. doi: 10.1182/blood-2012-06-435297 PMC3471515 22859607

[94] Gorodetskiy V Probatova N Sidorova Y Kupryshina N Obukhova T Vasilyev V. The non-Leukemic T Cell Large Granular Lymphocytic Leukemia Variant With Marked Splenomegaly and Neutropenia in the Setting of Rheumatoid Arthritis - Felty Syndrome and Hepatosplenic T Cell Lymphoma Mask. Am J Blood Res (2021) 11:227. 34322285 PMC8303016

[95] Schwaneck EC Renner R Tony HP Weber A Geissinger E Gernert M. Clonal Expansion of Large Granular Lymphocytes in Patients With Spondyloarthritis and Psoriatic Arthritis Treated With Tnfα Inhibitors. Rheumatol Int (2021) 41:1979–86. doi: 10.1007/S00296-021-04872-W 33991197

[96] Pro B Allen P Behdad A. Hepatosplenic T-Cell Lymphoma: A Rare But Challenging Entity. Blood (2020) 136:2018–26. doi: 10.1182/BLOOD.2019004118 PMC7596851 32756940

[97] Thai A Prindiville T. Hepatosplenic T-Cell Lymphoma and Inflammatory Bowel Disease. J Crohns Colitis (2010) 4:511–22. doi: 10.1016/J.CROHNS.2010.05.006 21122554

[98] Romero V Fert-Bober J Nigrovic PA Darrah E Haque UJ Lee DM. Immune-Mediated Pore-Forming Pathways Induce Cellular Hypercitrullination and Generate Citrullinated Autoantigens in Rheumatoid Arthritis. Sci Transl Med (2013) 5:209ra150. doi: 10.1126/SCITRANSLMED.3006869 PMC4032227 24174326

[99] Konig MF Andrade F. A Critical Reappraisal of Neutrophil Extracellular Traps and NETosis Mimics Based on Differential Requirements for Protein Citrullination. Front Immunol (2016) 7:461. doi: 10.3389/FIMMU.2016.00461 27867381 PMC5095114

[100] Konig MF Abusleme L Reinholdt J Palmer RJ Teles RP Sampson K. Aggregatibacter Actinomycetemcomitans-Induced Hypercitrullination Links Periodontal Infection to Autoimmunity in Rheumatoid Arthritis. Sci Transl Med (2016) 8:369ra176. doi: 10.1126/SCITRANSLMED.AAJ1921 PMC5384717 27974664

[101] Romero V Darrah E Andrade F. Generation of Distinct Patterns of Rheumatoid Arthritis Autoantigens by Peptidylarginine Deiminase Types 2 and 4 During Perforin-Induced Cell Damage. Arthritis Rheumatol (Hoboken NJ) (2020) 72:912–8. doi: 10.1002/ART.41196 PMC7255925 31876120

[102] Andrade F Roy S Nicholson D Thornberry N Rosen A Casciola-Rosen L. Granzyme B Directly and Efficiently Cleaves Several Downstream Caspase Substrates: Implications for CTL-Induced Apoptosis. Immunity (1998) 8:451–60. doi: 10.1016/S1074-7613(00)80550-6 9586635

[103] Casciola-Rosen L Andrade F Ulanet D Wong WB Rosen A. Cleavage by Granzyme B Is Strongly Predictive of Autoantigen Status: Implications for Initiation of Autoimmunity. J Exp Med (1999) 190:815–25. doi: 10.1084/JEM.190.6.815 PMC2195625 10499920

[104] Darrah E Rosen A. Granzyme B Cleavage of Autoantigens in Autoimmunity. Cell Death Differ (2010) 17:624–32. doi: 10.1038/CDD.2009.197 PMC3136751 20075942

[105] Takizawa Y Sawada T Suzuki A Yamada R Inoue T Yamamoto K. Peptidylarginine Deiminase 4 (PADI4) Identified as a Conformation-Dependent Autoantigen in Rheumatoid Arthritis. Scand J Rheumatol (2005) 34:212–5. doi: 10.1080/03009740510026346-1 16134727

[106] Zhao J Zhao Y He J Jia R Li Z. Prevalence and Significance of Anti-Peptidylarginine Deiminase 4 Antibodies in Rheumatoid Arthritis. J Rheumatol (2008) 35:969–74. 18398945

[107] Halvorsen EH Pollmann S Gilboe IM van der Heijde D Landewé R Ødegård S. Molberg. Serum IgG Antibodies to Peptidylarginine Deiminase 4 in Rheumatoid Arthritis and Associations With Disease Severity. Ann Rheum Dis (2008) 67:414–7. doi: 10.1136/ARD.2007.080267 18006540

[108] Harris ML Darrah E Lam GK Bartlett SJ Giles JT Grant AV. Association of Autoimmunity to Peptidyl Arginine Deiminase Type 4 With Genotype and Disease Severity in Rheumatoid Arthritis. Arthritis Rheum (2008) 58:1958–67. doi: 10.1002/ART.23596 PMC2692635 18576335

[109] Darrah E Kim A Zhang X Boronina T Cole RN Fava A. Proteolysis by Granzyme B Enhances Presentation of Autoantigenic Peptidylarginine Deiminase 4 Epitopes in Rheumatoid Arthritis. J Proteome Res (2017) 16:355–65. doi: 10.1021/ACS.JPROTEOME.6B00617 PMC5218978 27700100

